# A Multiscale Ex Vivo Method to Investigate Intervertebral Disc Strain and Fiber Recruitment in Anterolateral Bending Using 9.4T MRI‐DVC and DIC Microscopy

**DOI:** 10.1002/jsp2.70154

**Published:** 2025-12-25

**Authors:** T. D. Slater, K. A. Raftery, V. M. van Heeswijk, A. Thambyah, N. Newell

**Affiliations:** ^1^ Department of Bioengineering Imperial College London London UK; ^2^ Department of Chemical and Materials Engineering University of Auckland Auckland New Zealand

**Keywords:** annulus fibrosus, anterolateral bending, DIC microscopy, ex vivo, fiber recruitment, microstructural analysis, MRI DVC, multiscale biomechanics, porcine intervertebral disc, strain distribution

## Abstract

**Study Design:**

Ex vivo, multiscale analysis of disc strain using ultrahigh‐field MRI‐based digital volume correlation (MRI‐DVC) and differential interference contrast (DIC) microscopy.

**Objective:**

To evaluate the relationship between three‐dimensional strain distributions and collagen fiber recruitment in porcine cervical intervertebral discs under flexion and lateral bending.

**Summary of Background Data:**

Flexion combined with lateral bending is often linked to disc herniation, yet the strain patterns and fiber‐level changes in the annulus fibrosus are not well understood. Multiscale characterization is essential to uncovering failure mechanisms.

**Methods:**

Four porcine cervical motion segments were scanned in neutral and anterolaterally (AL)‐bent postures using 9.4T MRI, with 3D strains calculated via DVC. Samples were sectioned and imaged with DIC microscopy to quantify collagen fiber recruitment based on fiber crimp patterns, using a crimp grading scale (0 = fully straight, 1 = semi‐crimped, 2 = uncrimped).

**Results:**

MRI‐DVC revealed an inhomogeneous strain distribution in AL‐bent discs, with higher magnitudes compared to the neutral discs. Fiber uncrimping was greater in the AL‐bent discs (mean crimp grade: 0.44, mostly straight) compared with the neutral discs (1.56, predominantly crimped). Across the bending axis, the anterior‐right region exhibited higher strains than the posterior‐left (minimum principal strain ~25% greater), which correlated with the presence of sequential lamellae having straight and fully‐crimped fibers. A greater amount of fiber uncrimping was observed in the posterior‐left than anterior‐right disc regions.

**Conclusion:**

This study confirms the suitability of MRI‐DVC combined with DIC microscopy for relating macroscopic strains to microscopic fiber crimp, and for identifying regions of high strain across multiple length scales. Under AL‐bending, this methodology revealed that the disc's posterior region exhibited taut fibers, which may contribute to its susceptibility to herniation.

## Graphical abstract

Ultrahigh‐field MRI‐based digital volume correlation combined with optical DIC microscopy enables direct linkage of three‐dimensional disc strain fields with collagen fiber recruitment across multiple length scales. Applied to anterolaterally bent porcine cervical discs, these multiscale approaches revealed pronounced regional strain heterogeneity and differential fiber uncrimping across adjacent annular lamellae. Regions experiencing elevated strain exhibited taut posterior annular fibers, demonstrating how mechanical asymmetries under combined bending may predispose the disc to herniation.

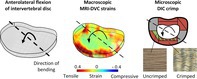

## 1 Introduction

Intervertebral disc disorders are a major contributor to chronic lower back pain, affecting millions worldwide and constituting the primary non‐fatal health burden globally [1, 2]. Understanding the mechanical behavior of intervertebral discs under physiological loading is essential for developing effective treatments and preventative measures. Disc degeneration can increase strains within the disc by up to 43%, which may increase the risk of structural disc failure [3]. Therefore, accurate mapping of strain distributions within discs is critical for identifying the regions most at risk of injury.

The intervertebral disc consists of three main components: the annulus fibrosus (AF), composed of concentric lamellae (made of collagen fiber bundles), the nucleus pulposus (NP), a proteoglycan‐rich, hydrated core that generates hydrostatic pressure under load [4], and the endplates. When subjected to loading, the disc itself becomes strained to accommodate movement. Such macroscale strains translate down to the microstructure and to the collagen fiber bundles. Microstructural studies have shown that collagen fiber bundles in tissues are naturally crimped, but straighten when the bulk tissue is under tension [5, 6].

Flexed postures are commonly used in ex vivo disc herniation models to simulate real‐life loading scenarios and investigate disc vulnerability [7, 8, 9]. These postures mimic common movements, such as bending to pick up objects [8, 9], or are used to overstress the disc to expose its regions that are vulnerable to failure [10, 11]. Under flexion, the posterior region of the intervertebral disc experiences the least axial compression [12], the greatest tensile deformation [12], and the highest shear strain [13]. This resulting strain environment may drive a multiscale tissue response that reflects an increased mechanical vulnerability in the posterior region of the disc.

A multiscale understanding of strain is essential to reveal how the intervertebral disc responds to mechanical loading, from whole‐tissue deformation down to the behavior of individual fiber bundles within the AF. While macroscale strain mapping can identify regions of high deformation and potential failure [3], such methods alone do not explain how loading is distributed among the complex fiber microarchitecture of the AF. The natural uncrimped state of collagen fibers, together with the cross‐ply architecture of the bundles, suggests preferential recruitment of fibers during bulk tissue loading. Measuring strain at this level can highlight how local fiber architecture is affected in regions under high or low macroscopic strain. Conversely, microscale changes are highly localized, so without a broader macroscopic strain map, it is difficult to interpret their significance within the whole disc. Therefore, a method for measuring strains at multiple length scales is needed to create a direct link between global, macro strain patterns and localized microstructural responses, thus offering a more complete, multiscale understanding of the mechanical processes that occur in vivo.

Combining non‐invasive ultrahigh‐field MRI with digital volume correlation (DVC) and differential interference contrast (DIC) microscopy enables strain analysis across multiple length scales, supporting a multiscale understanding. MRI‐DVC quantifies three‐dimensional strain distributions in intact samples, providing a whole disc, macroscopic view of deformation under load [14, 15, 16]. Because this method is non‐destructive, the same sample can then be used for further testing and imaging, allowing direct linkage between global strain patterns and microstructural behavior. Following MRI‐DVC, DIC microscopy (requiring sectioning of the disc) may be used to visualize and quantify collagen fiber uncrimping within the AF at a microscopic level. This method has previously been applied to measure fiber strains and local deformation, making it ideal for capturing the micro‐scale mechanics of collagen fiber recruitment [6].

Therefore, the first aim of this study was to evaluate a method for taking multiscale measurements of strain, using MRI‐based digital volume correlation (MRI‐DVC) and differential interference contrast (DIC) microscopy. These two techniques were used to measure strain in the intervertebral disc, focusing on macroscale and microscale deformation, respectively. The second aim was to investigate whether patterns in strain could provide preliminary insights into why the posterior AF is mechanically vulnerable to herniation under physiological loading.

## 2 Materials and Methods

### 2.1 Specimen Preparation

Four cervical (C2–C3 and C3–C4) vertebra‐disc‐vertebra functional spine units (FSU) were obtained from two adult‐sized pigs. The vertebrae retained growth plates, consistent with animals that were mature but not fully skeletally fused. From each FSU, the facet joints and ligaments were removed, except for the anterior and posterior longitudinal ligaments. Throughout the preparation, FSUs were regularly sprayed with phosphate buffered saline (PBS, 0.15 mol L^−1^). Both vertebrae were embedded in cylindrical pots with PMMA (bone cement) to a depth of 1 cm, with the disc centre visually aligned to the centre of the cup in the transverse plane. Freeze–thaw cycles were limited to a maximum of two per disc.

### 2.2 Loading Apparatus

**FIGURE 1:**
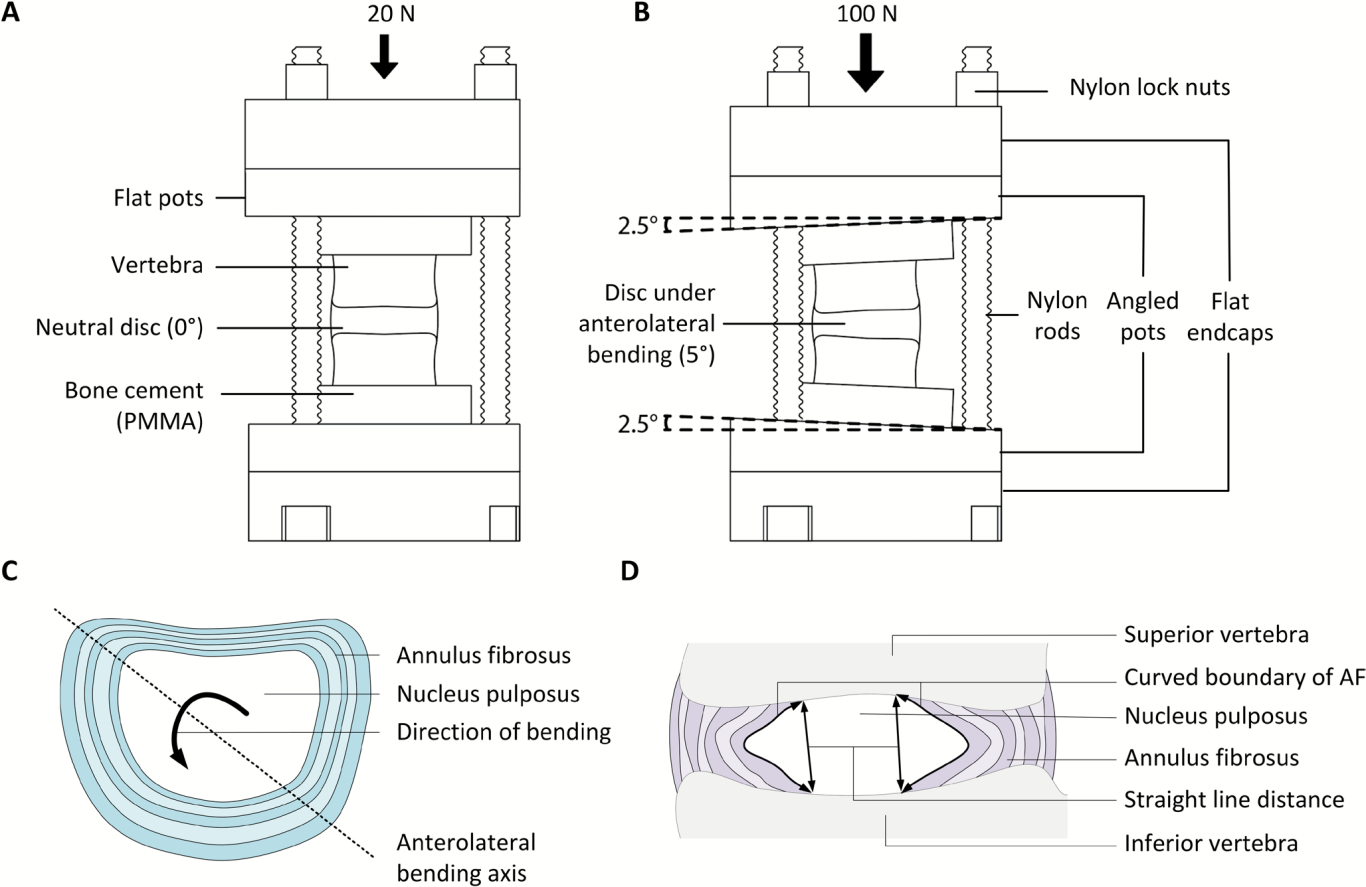
Diagram of the MRI‐compatible loading apparatus showing the potted disc in (A) the neutral (0°) configuration and (B) at a 5° AL‐bend. (C) Transverse plane diagram of the disc depicting the AL bending axis. (D) Mid‐coronal plane diagram showing lamellar curvature measurements, calculated as the ratio of the traced curved AF boundary length to the straight‐line distance between its endpoints.

A custom‐made MRI‐compatible rig was designed to apply an axial load and to position each functional spinal unit (FSU) in either a neutral posture or a right anterolateral (AL) bend of 5° (Figure 1). The rig consisted of two end caps, adjacent to interchangeable mounting plates (flat or angled), and connected by nylon rods. Each plate contained recessed cut‐outs designed to hold the PMMA pots securely in place and restrict movement. Each sample was scanned twice while either in its neutral or AL‐bent posture (Table 1).

**TABLE 1:** Discs tested within the study, including animal ID, disc level, geometric measurements, and the loading configuration (angle, load) applied during each of the two MRI scans performed on each specimen.

Disc	Animal ID	Disc level	Disc height (mm)	Anterior–posterior disc width (mm)	Medial‐lateral disc width (mm)	Scan 1	Scan 2
1	1	C2–C3	4.6	20.5	28.3	Neutral (0°, 20 N)	AL‐bent (5°, 100 N)
2	2	C3–C4	5.2	20.5	32.1	Neutral (0°, 20 N)	AL‐bent (5°, 100 N)
3	2	C2–C3	4.4	20.2	33.3	Neutral (0°, 20 N)	Neutral (0°, 20 N)
4	1	C3–C4	5.1	20.6	32.1	Neutral (0°, 20 N)	Neutral (0°, 20 N)

For the scan in the neutral posture, the potted FSU was placed into both the inferior and superior recessed flat plates, and an axial load of 20 N was applied using a materials testing machine (Instron 5584, Norwood, MA, USA), sufficient to hold the potted‐FSU in place. The load was maintained for 1 h before MRI scanning to counter any post‐mortem swelling. After 1 h, nuts were threaded onto the rods to secure the discs in place, maintaining a constant displacement and preventing movement during scanning. A 1 h preload has previously been shown to be sufficient to minimize internal tissue stress relaxation and is suitable for MRI‐DVC measurements [14].

For the scan while bent anterolaterally, the FSU was placed into the recess of the inferior angled plate (angled at 2.5°), with the anterolateral corner of the disc positioned in the direction of maximum gradient. The superior angled plate was then lowered, and the disc was manually deformed to insert the upper pot into the recess (also angled at 2.5°), causing the disc to bend a total of 5° about the AL‐axis. This singular bend was mechanically equivalent to a simultaneous application of 3.5° flexion and 3.5° lateral bending. An axial load of 100 N was applied for 1 h, after which the displacement was fixed (as above).

### 2.3 MRI Protocol

Following sample placement into the loading rig, discs were imaged using a 9.4T ultra‐high field MRI scanner (Bruker BioSpin MRI GmbH, Ettlingen, Germany). The scan was a T_2_‐weighted, TurboRARE (Rapid Acquisition with Relaxation Enhancement) sequence, with a coronal plane resolution of (90 × 90) μm, a slice thickness of 800 μm, and a ~30 min scan time. These settings were specially designed to allow DVC analysis to be conducted on lumbar intervertebral discs [3, 14, 15, 17, 18].

To investigate the effect of anterolateral bending on the disc strains, first an MRI scan was acquired in a neutral posture, followed by a scan in an anterolaterally bent posture. Control samples were scanned twice in the neutral posture to perform a zero‐strain study which quantified MRI‐DVC error by assuming no deformation between sequential, identical‐posture neutral scans [14, 19, 20].

### 2.4 Disc Geometry Measurements

Intervertebral disc height was measured on the mid‐sagittal MRI slice using the average height of three vertical lines positioned at the anterior, central, and posterior of the disc. Disc widths were measured from the midpoint of the disc height on axial reconstructions of the MR images. Anterior–posterior (sagittal) width was measured along the sagittal axis, while medial–lateral width was measured at the widest point of the disc, parallel to the medial–lateral axis. The FSUs were of similar size to those in other studies on porcine cervical vertebrae (Table 1) [21, 22].

### 2.5 Lamellae Curvature Measurements

Lamellar curvature was measured to determine whether macroscopic disc deformations arose from bending/straightening of the lamellae or from their stretching, providing an in‐plane assessment not captured by MRI‐DVC. Curvature was quantified from mid‐coronal MRI images using ImageJ (version 1.53 t). The three most central, coronal slices were first identified, ensuring the AF appeared parallel to the imaging plane. In each selected slice, the boundaries between NP and AF were then manually traced along the curved, NP‐AF contours. A straight line was then drawn between the superior and inferior endpoints of the traced boundaries (Figure 1D). Curvature was calculated as the ratio of the curved boundary length to the corresponding straight‐line distance. This procedure was repeated for each of the three slices, and the mean of these three values was used as the final curvature measurement for each disc.

### 2.6 Digital Volume Correlation (DVC) Protocol and Analysis

The three‐dimensional deformations within the intervertebral discs were assessed using DVC applied to both neutral and AL‐bent MRI scans, following the methodology described by Tavana et al. [14]. The process involves dividing the entire volumetric image into smaller subsets, where distinct internal tissue patterns from the unloaded image are tracked in the corresponding loaded image. Before running the DVC analysis, images were pre‐processed using bicubic interpolation to estimate the signal intensities between adjacent slices along the *z*‐axis [23], in order to convert the original non‐cubic voxel dimensions (90 × 90 × 800 μm^3^) into cubic voxels (90 × 90 × 90 μm^3^) using ImageJ 1.49μ (National Institutes of Health, USA). To isolate the disc, each sample was manually segmented in Mimics (Materialize HQ, v.19.0, Leuven, Belgium) to create a binary mask excluding bone and surrounding tissues.

DVC analysis was performed using DaVis 10.2.1 (LaVision, Germany), employing a combined Fast Fourier Transform and Direct Correlation (FFT + DC) approach. Strains were computed as finite Lagrangian strains, allowing quantification of large deformations over the subvolume. Previous work has shown that FFT + DC is an effective method for this type of analysis and that a subset size of 48 × 48 × 48 voxels with 50% overlap offers a good compromise between random strain error (~1%) and spatial resolution (2.16 mm) [14]. DaVis software was used to calculate axial, maximum shear, maximum (MaxPS), and minimum (MinPS) principal strain components, while radial and circumferential strains were derived in MATLAB (MathWorks Inc., Natick, MA) [14, 15]. The radial and circumferential strains were calculated by defining a local coordinate system for each subset based on a line drawn from the disc centroid to its boundary. The intersection point determined the local tangential (circumferential) and normal (radial) directions [15, 16].

MATLAB was also used to generate three‐dimensional heatmap visualizations and perform regional analysis of the discs. The discs were split into two regions, across the bending axis, herein referred to as the anterior‐right and posterior‐left regions. The mean and standard deviations were calculated from the grouped strain values; however, any strain values associated with low correlation coefficients (< 0.8), as calculated by DaVis, were excluded.

### 2.7 Macro Strain Measurement Error

The subset size determines the resolution of the strain field and also influences the measurement error. In this study, a voxel size of 0.9 mm and a subset size of 48 × 48 × 48 voxels were used, corresponding to an effective spatial resolution of approximately 43 mm. To quantify the measurement error, a zero‐strain analysis was performed on Disc 3 and Disc 4 under the assumption that no strain was present. For the maximum principal strain, the mean absolute error (MAER) and the standard deviation of the error (SDER) were calculated to evaluate the measurement accuracy for the MRI‐DVC measurements for this study.

### 2.8 Sectioning and Microscopic Imaging Procedure

Following MR imaging, each FSU was fixed in 4% neutral buffered formalin for 1 week while held in the respective neutral or AL‐bent posture. Each FSU was then decalcified in 10% formic acid for 2 weeks [7]. The macro deformations (7.5° AL‐bend) were manually verified by inclinometer following fixation, in the absence of external load, confirming that the overall sample deformation was retained throughout the fixation process.

**FIGURE 2:**
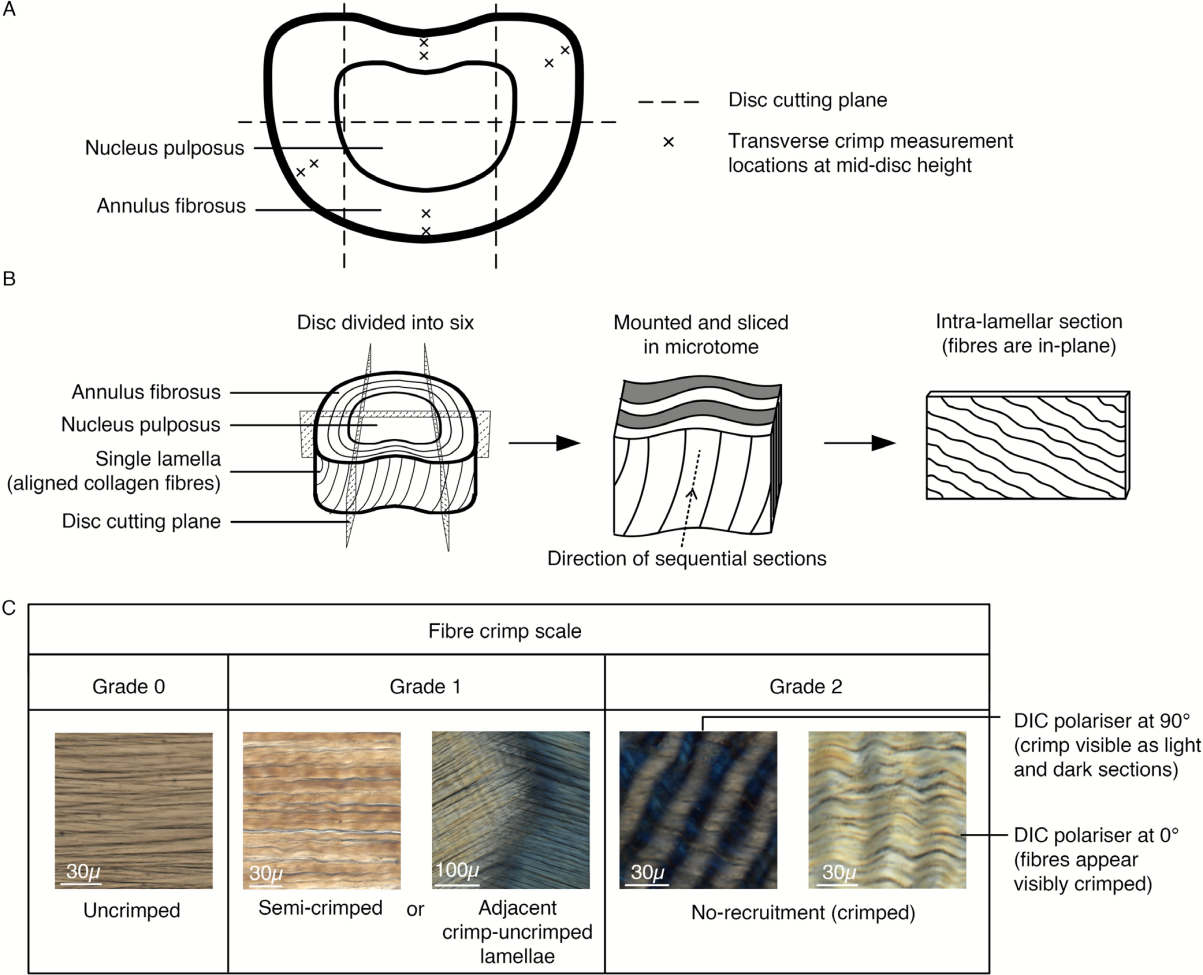
(A) Transverse view with dotted lines showing the axes of the planar cuts, and crosses where crimp was measured. (B) Diagram of disc segmentation, the sectioning‐plane, and the alignment with in‐plane fibers. (C) Fiber recruitment scale (0–2), with representative images used to guide crimp grading.

Based on the MRI‐DVC analysis, the disc regions that experienced the maximum and minimum strains under loading were to be assessed with DIC microscopy. To facilitate this, each FSU sample was cut into six smaller blocks and serially sectioned into 30 μm thick slices (Figure 2A,B). Slices were taken aligned to the direction of the lamellae. The sections were wet‐mounted and imaged using a Nikon Eclipse Ni microscope (Nikon Instruments Inc., Tokyo, Japan) equipped with a DS‐Ri 2 camera, a 10× objective lens, and DIC prisms (Figure 2C).

### 2.9 Fiber Recruitment Analysis

Fiber recruitment was quantitatively assessed in defined regions of the AF to evaluate the microstructural response under load (Figure 2A). The AF was divided into outer, mid, and inner thirds, though only the outer and mid‐AF were analyzed due to insufficient in‐plane tissue within the inner AF. For each region, recruitment was scored separately in the outer and mid‐AF at the mid‐disc height of the three most central slices. This approach provided a consistent, regionally comparable metric, as crimp spacing measurements can vary with radial collagen composition [24, 25] and become unreliable where fibers are fully uncrimped.

A visual scale from 0 (uncrimped, or fully straightened) to 2 (crimped) was applied (Figure 2C), following the methodology of Zhao et al. [6]. The three individual scores within each region were then averaged to produce a mean crimp grade. Where adjacent lamellae showed differences in crimp, such as fully crimped (2) next to uncrimped (0) fibers, a score of 1 was assigned to indicate partial recruitment. To assess the inter‐observer reliability of the crimp grading, all measurements were repeated independently by TS and KR. In cases of disagreement, a consensus was reached between observers.

## 3 Results

Strain patterns and fiber crimp differed between the AL‐bent discs and the control discs. Ultra‐high‐field MRI‐DVC and DIC imaging revealed distinct deformation profiles and fiber arrangements consistent with their respective loading postures.

### 3.1 MRI‐DVC

**FIGURE 3:**
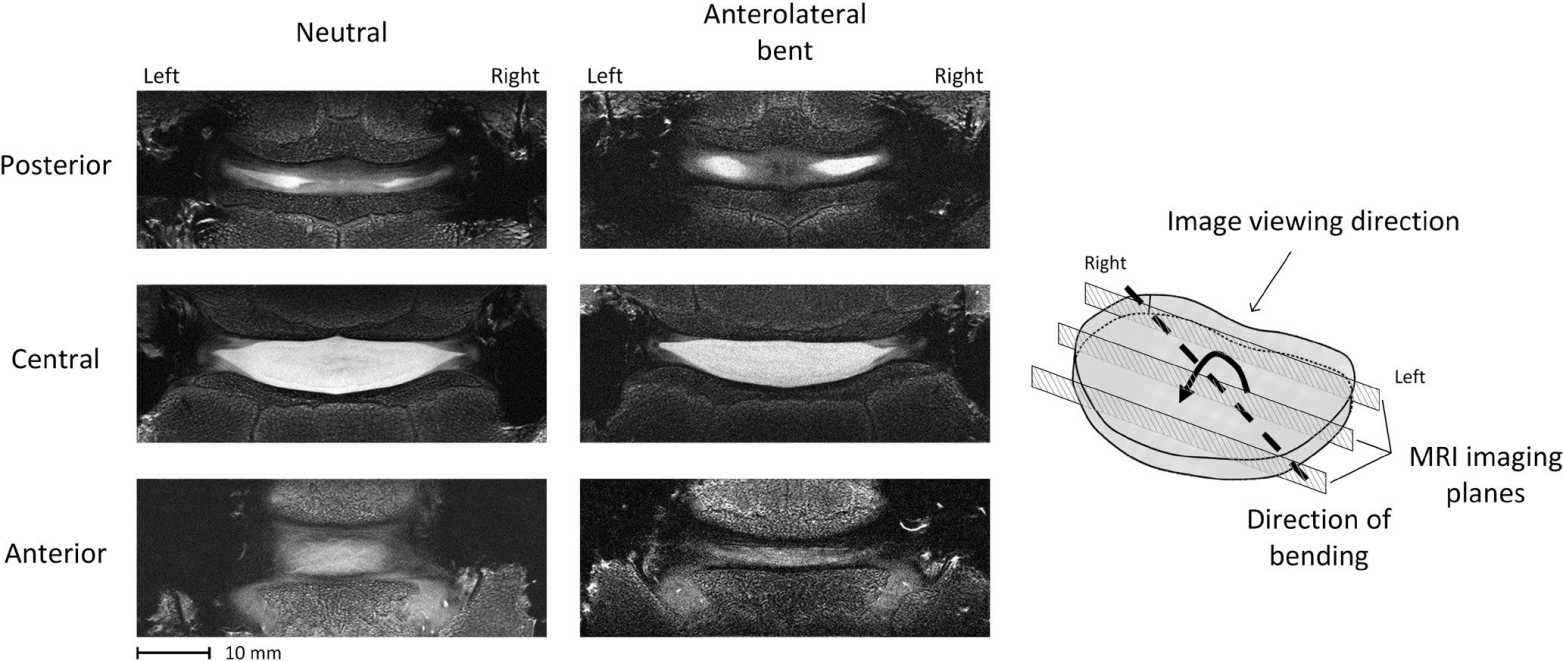
MRI scans of Disc 1 showing coronal slices at the posterior, central, and anterior regions of the disc, in both the neutral position and after applying a 5° anterolateral bend. Using tangents to the superior and inferior endplates to define the angle, it was confirmed that the achieved bending was (3.4 ± 0.6)°, consistent with the targeted 3.5° orientation in the coronal plane.

MRI scans revealed a bright NP, with some surrounding AF visible, suitable for digital volume correlation (DVC) analysis (Figure 3). During AL bending, the inner AF showed asymmetric curvature changes. On the right‐hand side, mean curvature increased by (24.4 ± 5.4)%, while the left‐hand side increased by only (7.5 ± 3.4)% (Table 2). These changes were driven by a reduction in the disc‐height component (−15.5 ± 7.7)%, with inner lamellae length remaining largely unchanged (< 3% change).

**TABLE 2:** Intervertebral disc curvature measurements for left and right sides during two scans (neutral and AL‐bent postures), with standard deviations.

Disc	Category	Curvature scan 1 (neutral posture) ± standard deviation		Curvature scan 2 (neutral or AL‐bent posture) ± standard deviation		Percentage change (%) ± standard deviation	
		Left	Right	Left	Right	Left	Right
1	AL‐bent	3.0 ± 0.05	2.8 ± 0.10	3.2 ± 0.16	3.3 ± 0.04	5.7 ± 5.6	15.5 ± 4.1
2	AL‐bent	2.5 ± 0.07	3.0 ± 0.06	2.7 ± 0.06	4.0 ± 0.3	9.2 ± 3.8	33.3 ± 9.9
3	Neutral	2.4 ± 0.09	2.3 ± 0.07	2.3 ± 0.05	2.3 ± 0.08	1.7 ± 4.4	0.3 ± 4.7
4	Neutral	3.0 ± 0.15	3.0 ± 0.13	3.0 ± 0.2	3.1 ± 0.3	2.1 ± 9.2	3.8 ± 10.5

**FIGURE 4:**
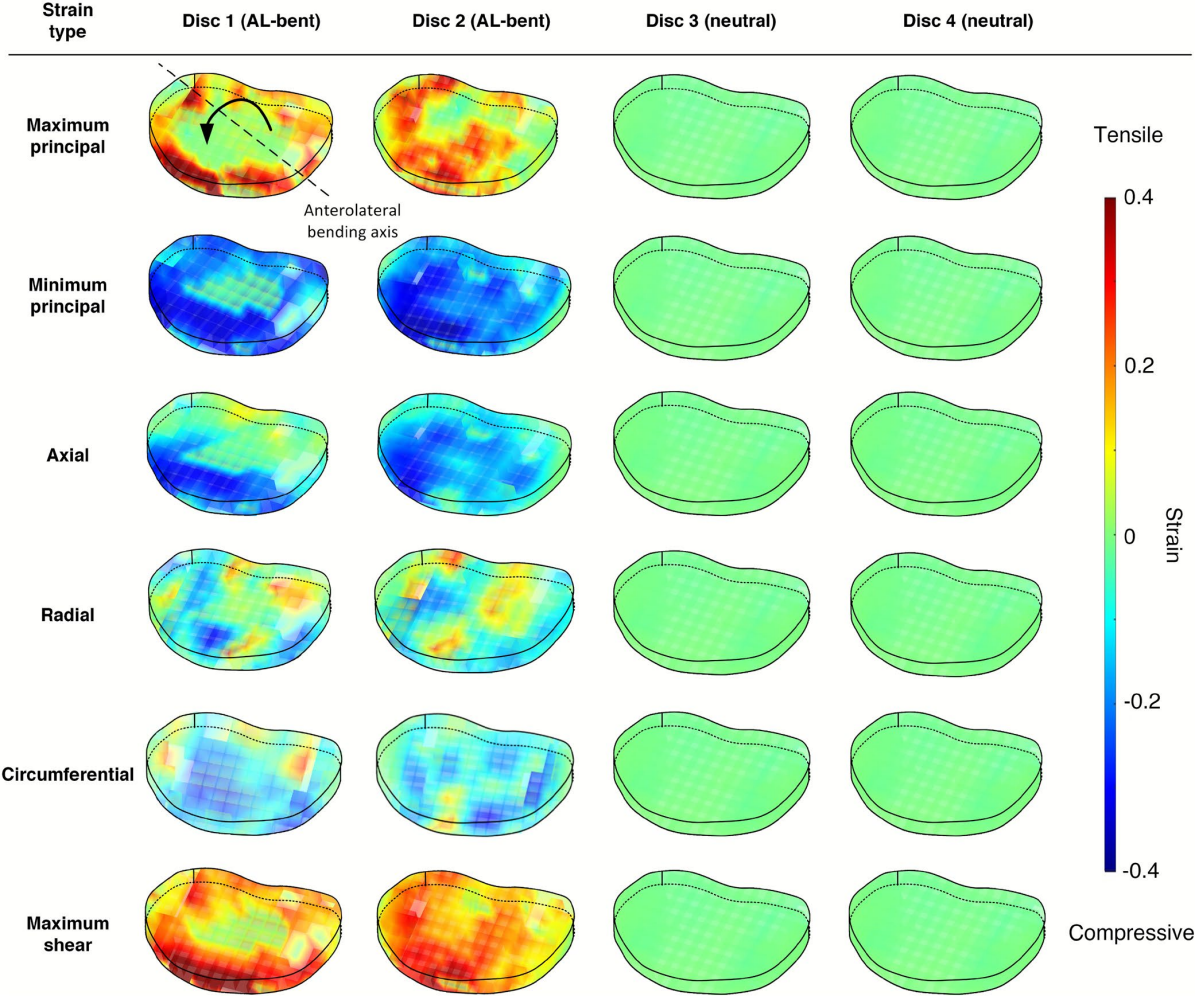
Three‐dimensional strain maps derived from MRI‐DVC analysis, showing six strain components: maximum principal, minimum principal, maximum shear, axial, radial, and circumferential. A reference AL‐bending axis has been added to the maximum principal strain map of Disc 1.

AL‐bent discs showed heterogeneous strain patterns across all strain types (Figure 4). The mean MinPS strain values for Disc 1 and Disc 2 were (−21.9 ± 7.7)% and (−25.1 ± 7.2)%, respectively, while the mean MaxPS values were (14.3 ± 12.2)% and (20.2 ± 18.5)%.

**FIGURE 5:**
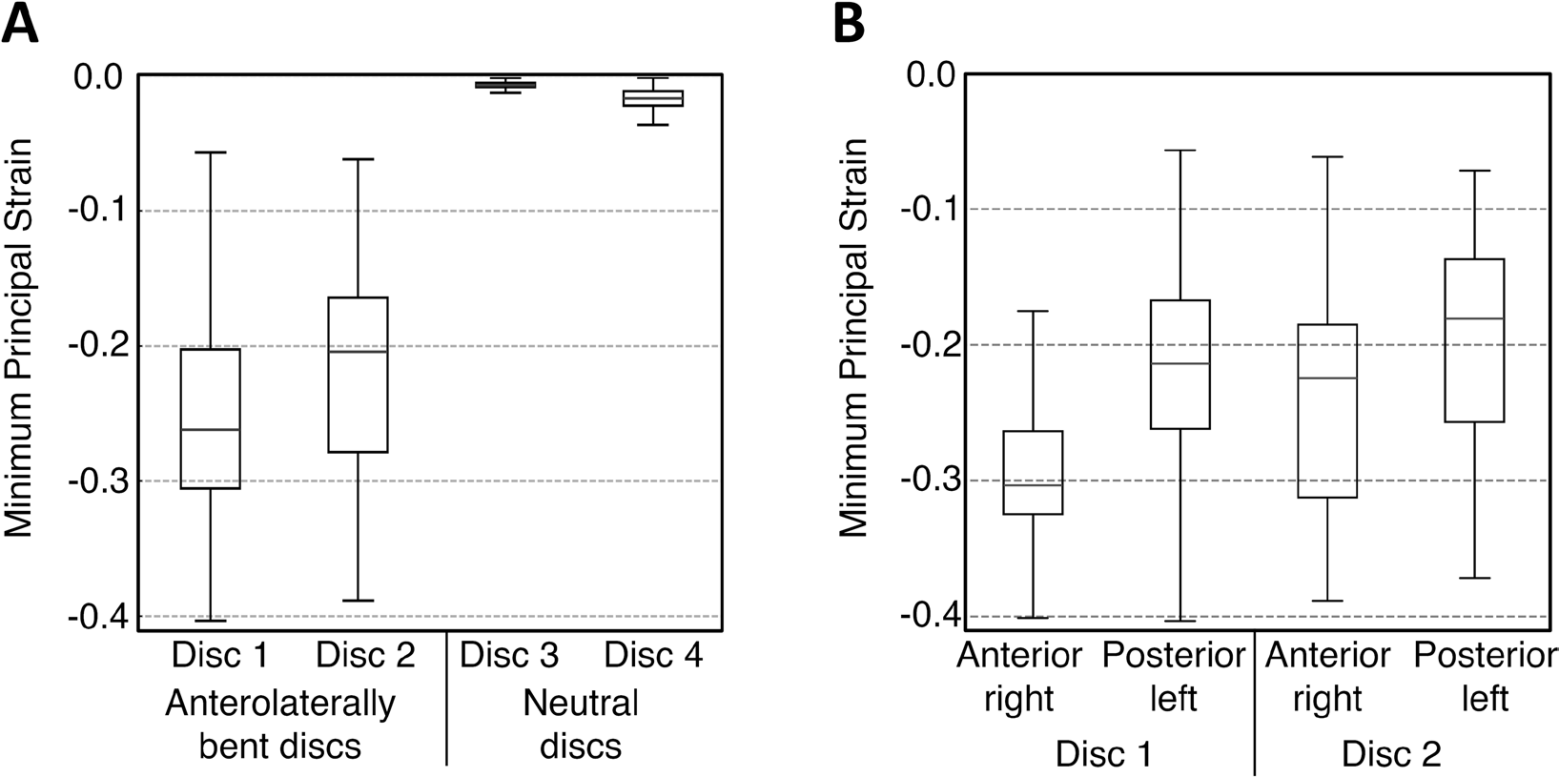
(A) Boxplots showing MinPS for all four intervertebral discs. (B) Comparison of strain distribution between anterior‐right and posterior‐left regions in the AL‐bent discs. For all types of strain and both regions, additional boxplots are provided in Appendix A.

Multi‐axial strain distributions were observed in both discs across all directions (Figure 5A, Appendix A). Across the bending axis, the macroscopic MinPS strain magnitudes were greater in the anterior‐right (averaging −26.4%) than the posterior‐left region (averaging −20.6%) (Figure 5B). The strain values in the neutral posture discs were considerably lower than strains measured in the AL‐bent discs, with a MinPS of (−0.6 ± 0.2)% in Disc 3 and (−1.8 ± 1.1)% in Disc 4. Zero‐strain scans showed the largest MAER in Disc 4, at 2353 microstrain (0.235%), and the associated SDER was 954 (0.095%), indicating that strains greater than ~0.4% can be reliably detected.

### 3.2 Fiber Crimp

**FIGURE 6:**
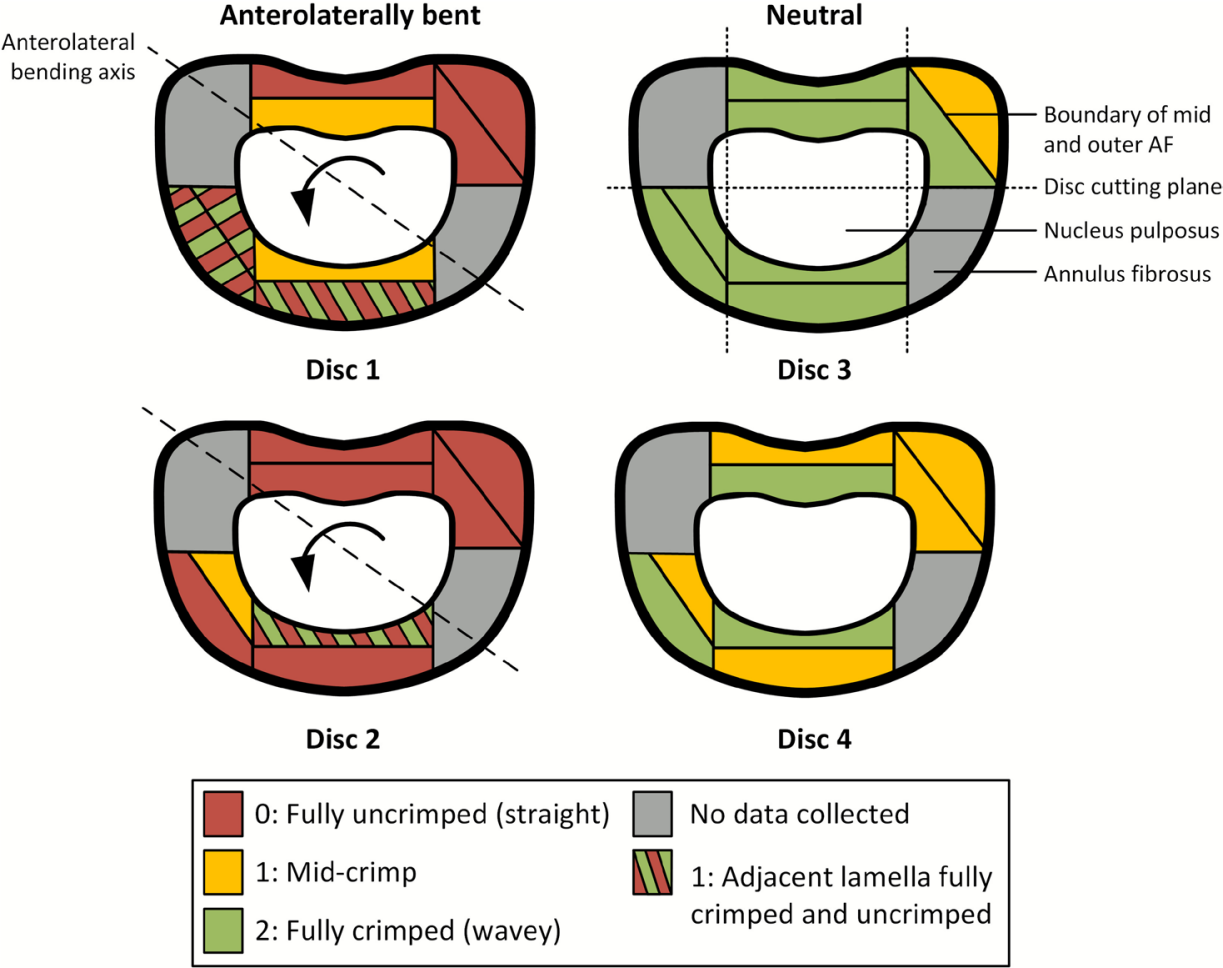
Mean fiber recruitment across transverse sections of intervertebral discs, highlighting regions of fully crimped, semi‐crimped, and uncrimped fibers under AL‐bent and neutral postures. Boundaries within the AF indicate the division between mid and outer AF regions.

Individual fiber crimp measurements within a single sampling area were highly consistent across the three microscopic slices, with identical grades in 88% of areas (28/32). In the remaining 12% of areas, the differences never exceeded one grade point, indicating minimal intra‐disc variation. The inter‐observer reliability was found to be 85%, with all disagreements being no greater than one grade. AL‐bent discs showed substantial uncrimping compared to neutral discs, with mean crimp grades of 0.44 (56% of measurements graded 0) versus 1.56 (63% of measurements graded 2), respectively (Figure 6). In the neutral posture, only 6% of regions were fully uncrimped, compared to 81% in AL‐bent discs. Conversely, 94% of regions in neutral discs remained fully or semi‐crimped (19% in AL‐bent discs). Additionally, adjacent crimped and uncrimped lamellae were observed only in the anterior region of the AL‐bent discs. Across the bending axis, fibers in the posterior‐left region were more uncrimped (mean grade 0.13; 87.5% graded 0) than those in the anterior‐right (mean grade 0.75; 75% graded 1). Microscopic images and associated crimp values for all discs are presented in Appendix B.

### 3.3 MRI‐DVC and Fiber Crimp Comparison

**FIGURE 7:**
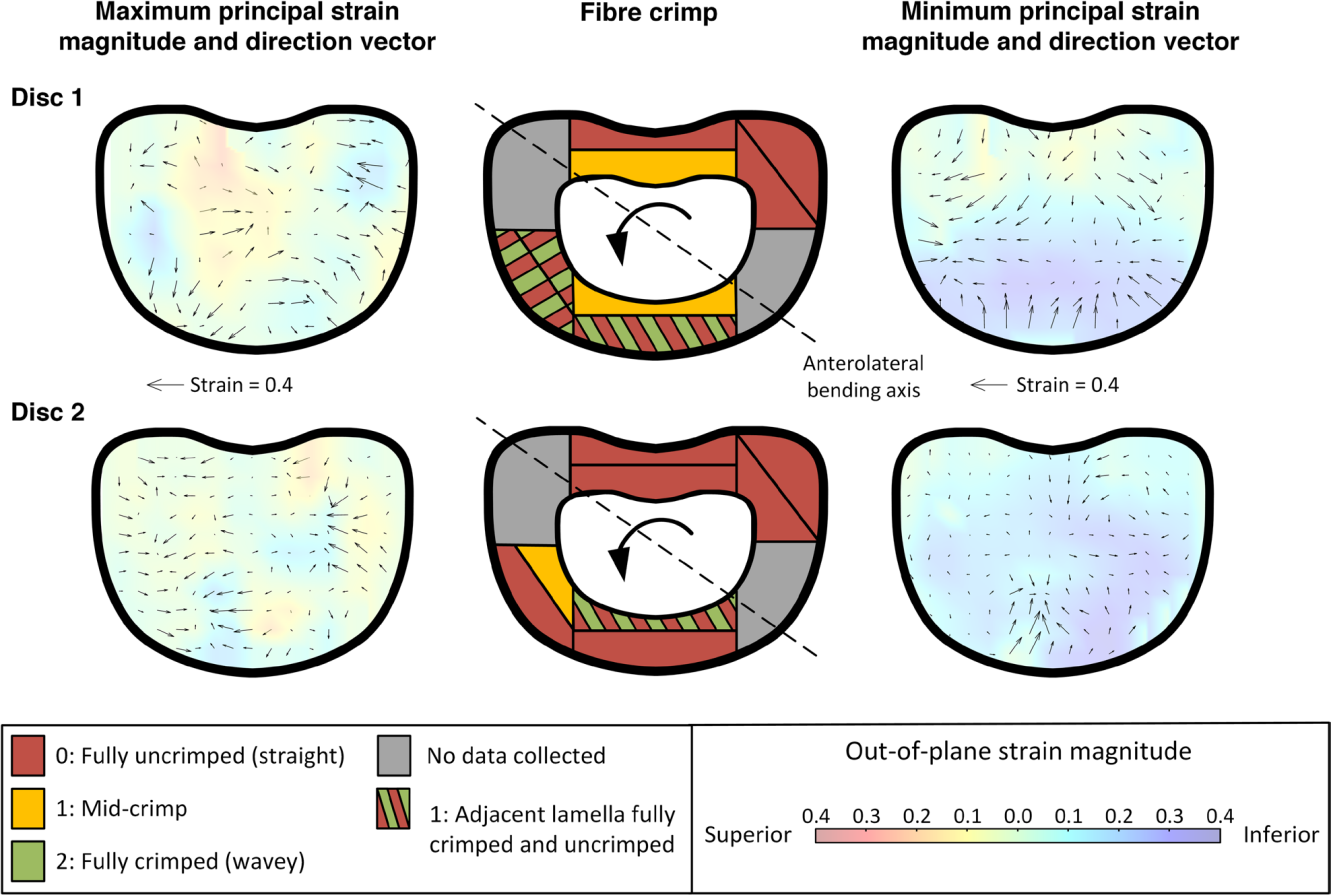
Principal strain (PS) directions and fiber crimp patterns across AL‐bent discs (Disc 1 and Disc 2). Maximum and minimum principal strain magnitudes and directions are shown as vectors (left, right), alongside corresponding fiber crimp (center). The axial component of the principal strains is overlaid as a heatmap to capture out‐of‐plane strain variations.

Fully uncrimped fibers (i.e., having a straight morphology) were not observed anywhere in the control discs, which experienced minimal loading and strain. In contrast, straight and partially uncrimped fibers were present in all assessed regions of the AL‐bent discs (MinPS between −5% and −40%), thus: uncrimping begins below this strain range and complete uncrimping occurs at strains between −5% and −40% (Figure 7).

## 4 Discussion

This study shows that collagen fiber crimp within the AF responds systematically to bending‐induced strains in the intervertebral disc, linking macro‐level deformation to microstructural behavior. Using a combined MRI‐DVC and DIC microscopy approach, we observed in two discs that the posterior‐left AF with relatively low tensile strains (Figure 5B, Appendix A) exhibited substantial fiber uncrimping (Figure 6), indicating localized tension within the fibers. Furthermore, the findings indicate that the macrostrain required for full fiber uncrimping is relatively low (~5%) compared to the peak strains observed (~40%).

### 4.1 Linking Macroscopic Strains to Microscopic Fiber Crimp

In neutral discs, the fibers remained mostly crimped (mean grade 1.63), indicating minimal uncrimping in the absence of strain. Conversely, AL‐bent discs were substantially uncrimped (0.44), even in the regions experiencing relatively low strains. Among the strain components measured, only MinPS showed complete separation between neutral and AL‐bent discs: neutral discs exhibited MinPS values close to 0%, while all AL‐bent regions had strains below −5% (Appendix A). This lack of overlap suggests that a MinPS of approximately −5% may represent a lower threshold for initiating fiber uncrimping. These findings support a direct link between macroscopic strain and microscopic fiber recruitment, and further samples are needed to elucidate the exact relationship.

Perhaps unexpectedly, the regions with the most uncrimping (located in the posterior‐left) in both AL‐bent discs did not align with the highest macroscopic tensile and compressive strains, which were found in the anterior‐right. We believe this is due to tensile and compressive strains being misaligned with fiber orientation, which may result in regions where fibers only partially uncrimp or uncrimp sequentially across layers, or lead to reduced uncrimping because the tensile strains are not aligned with the fiber directions. Additionally, due to scale differences, the microscopic crimp measurements may not be representative of the average macroscopic strain field it is within.

The regions of crimped and uncrimped fibers in adjacent lamellae were consistently aligned with areas of elevated MaxPS and MinPS in the anterolateral portion of the disc (Figure 7). AL‐bending may promote differential engagement of fibers in this region, so that only a subset of the fibers bear the load and become straightened. Consequently, these regions may be more susceptible to elevated strain compared with areas where both oblique and counter‐oblique fibers are simultaneously engaged, such as in the posterior and posterolateral regions of the disc. This hypothesis is consistent with earlier reports showing that uneven fiber‐recruitment under flexed loading conditions can generate high stress differentials between the lamellae and ultimately lead to failure in these regions [26, 27].

### 4.2 Macroscale Strains

The DVC results revealed an inhomogeneous strain distribution throughout the AL‐bent discs. In both discs, the maximum tensile strains and maximum compressive strains were located in the anterior and anterolateral regions (Figure 4). This pattern is consistent with previous experimental studies on human cadaveric tissue, [12, 13]. And, the observed inhomogeneity aligns with earlier findings from similar investigations [3, 14] and in vivo MRI‐DVC studies [28]. However, it contrasts with the approximately linear gradient observed in finite element models under uniform compression and flexion [29], and two‐dimensional ex vivo tests [12, 13, 30]. This likely reflects the macroscopic effects of structural features such as lamellar merging or termination [31], as well as the additional strain complexities introduced by three‐dimensional analysis.

### 4.3 Microscale Strains

Intervertebral discs bent along the AL‐axis exhibited extensive fiber uncrimping, in contrast to the neutral discs, which had fully crimped fibers (Figure 6). The uncrimping during physiological bending was expected, as collagen fiber bundles crimp in order to accommodate the imposed deformation and facilitate movement [25, 32, 33]. While fiber uncrimping has been observed in other joints under physiological loads [6, 32, 34, 35, 36, 37], our findings extend this to the intervertebral disc.

Alternating crimp‐uncrimp patterns between adjacent lamellae (Figure 5) reveal that highly localized strain gradients are present, especially in the anterior regions of the disc. This supports previous finite‐element models [38] and experimental tests [39], which suggest adjacent lamellae can experience large differences in strain. In Disc 2, however, this pattern was confined to the inner‐anterior region, suggesting that the disc anatomy and biological variation influence the fiber crimp in addition to the applied loading. Therefore, despite similar macroscopic strain profiles between Disc 1 and Disc 2, variations in anatomical structure likely influence microscopic fiber crimp.

### 4.4 Tissue‐Level Disc Adaptations to Strain

Our multiscale measurements indicate that collagen fibers can become fully uncrimped at relatively low strain levels (~5%), suggesting that further deformation must be accommodated by other mechanisms. In two discs, peak strains of 20%–40% were measured via MRI‐DVC, exceeding the strain that can be explained by fiber uncrimping. As these deformations occurred under a physiological bending [40], the disc must accommodate the strain through alternative, non‐damaging mechanisms. O'Connell et al. proposed that the radial curvature of the annular lamellae partially facilitates this adaptation: as the disc is physiologically bent (e.g., under flexion or anterolateral bending), the curved lamellae straighten, allowing for disc movement without additional uncrimping of fibers [12]. In this study, however, this effect was not seen in lateral regions adjacent to the disc centre, under a 5° bend and minimal compression. However, a preliminary test conducted at 15° demonstrated that this mechanism does occur at the centre of the disc, and even showed a reversal in fiber bending direction, from outwards to inwards. Therefore, it is expected that the lamellae's radial curvature became straightened in the posterior regions of Disc 1 and Disc 2. This could not be confirmed, however, due to the posterior lamellae being oblique to the MRI‐imaging plane.

The lamellar straightening mechanism may become impaired with age or degeneration. Degeneration induced lamellar buckling [10, 18] and reduced nucleus pressurization [41] may limit lamellar straightening, shifting more load directly onto the collagen fibers and increasing the risk of failure in the posterior AF [12]. This hypothesis aligns with clinical data: 90% of disc herniations occur posteriorly or posterolaterally [42]. Therefore, future studies should aim to determine whether degenerate discs retain functional crimp and lamellar mechanics necessary to allow physiological movements.

### 4.5 Limitations

The sample size of this study was small, limiting generalisability, though it was sufficient to demonstrate the feasibility of these methods for multi‐scale strain analysis. A larger sample size is needed to refine strain thresholds and to investigate further how specific strain types and their direction relate to fiber uncrimping.

The axial load applied (100 N) is lower than physiological in vivo values during bending [43], and therefore does not fully replicate physiological disc loading [43, 44]. This compromise was necessary because higher loads (~250 N) caused poor MR signal in the anterior regions of the disc, preventing reliable MRI‐DVC analysis.

MRI‐DVC, while quantitative, uses large subset sizes that spatially average data, potentially obscuring local strain variations. While the nominal minimum strain resolution was the same in all axes due to the isotropic voxel size, the isotropic voxels were generated via interpolation from the original images. Consequently, the effective measurement error in the sagittal plane is higher than in the other two axes, and this should be considered when interpreting strain values, particularly for sagittal‐plane deformations.

Crimp analysis was limited to in‐plane lamellae, and due to disc curvature, only highly localized regions could be assessed. The need for sectioning also prevented further testing of intact discs. Crimp was measured using a discrete grading scale rather than a continuous angle measure [32], to ensure consistency, especially where both fine and coarse crimp coexisted [6]. In regions with adjacent crimped and uncrimped fibers, values were averaged to 1, which may underrepresent local strain gradients.

The discs were held at a fixed displacement for MR imaging and fixation. During this time, the collagen fibers will have undergone stress relaxation, potentially returning to a crimped fiber state. While porcine neck discs were chosen as a non‐degenerate model for lumbar human discs [21], they are smaller in size and have a lower AF:NP ratio [21, 45], and are stiffer [40], which may limit the direct applicability of the findings to human discs.

### 4.6 Clinical Implications

The developed method provides a framework for assessing microstructural strains within individual lamellae alongside macroscale disc strains. By capturing these hierarchical strain responses, future studies could explore how micro‐ and macro‐strains are related and whether regions experiencing higher strains are more susceptible to localized degeneration [46]. Such research may provide an explanation for annular damages within specific regions of the disc, providing mechanistic insight as to why disc herniation occurs [47]. Clinically, this approach could support the identification of movements or loading patterns that place certain disc regions at higher risk. Such insights could guide the identification of movements to avoid when injured, ultimately supporting more effective disc healing and further injury prevention.

## 5 Conclusions

This study demonstrates that moderate anterolateral (5°) bending induces complex, regionally variable strain patterns within disc, with complete fiber uncrimping occurring even in areas subjected to relatively low strain (~5%). The presence of uncrimped fibers in the posterior region, observed in both specimens despite lower average strain, may provide a preliminary indication of why the posterolateral region is vulnerable to disc herniation. These observations offer initial insight into the possible relationship between microstructural fiber behavior and macroscale strains. The method developed provides a framework for evaluating how different postures and loading patterns affect macroscopic disc strains and microstructural fiber strains, offering potential for the development of specific treatment strategies. Such insights could guide the identification of movements to avoid when injured, ultimately supporting more effective disc healing and further injury prevention.

## Author Contributions

T.D.S., A.T., and N.N. contributed to the conception and design of the study and to funding acquisition; T.D.S., K.A.R., and V.M.H. contributed to the acquisition of laboratory data; T.D.S. and K.A.R. performed data analysis; all authors contributed to the interpretation of the data; T.D.S. drafted the manuscript; all authors critically revised the manuscript; and N.N. coordinated the study. All authors read and approved the final manuscript.

## Funding

This work was made possible by the International Exchange Scheme funded by The Royal Society (ES\R1\241 497). Equipment from the EPSRC‐funded Injury and Reconstruction Biomechanics Test Suite (EP/V029452/1) was used as part of this study.

## Ethics Statement

The authors have nothing to report.

## Conflicts of Interest

The authors declare no conflicts of interest.

## Supporting information


**Appendix A:** Box plots for all measured strains.

**Appendix B:** Representative images of each area of disc and corresponding crimp grade.

## Data Availability

The data that support the findings of this study are available from the corresponding author upon reasonable request.
