# Supplementary material for: A Multiscale Ex Vivo Method to Investigate Intervertebral Disc Strain and Fiber Recruitment in Anterolateral Bending Using 9.4T MRI‐DVC and DIC Microscopy
Source: JOR Spine. 2025 Dec 25;8(4):e70154. doi: 10.1002/jsp2.70154 (PMC12740184; doi:10.1002/jsp2.70154)
Supplement: Supplementary file 1 — Appendix A: Box plots for all measured strains. [file JSP2-8-e70154-s001.docx]

Supplementary Appendix A: Box plots for all measured strains

|  | |
| --- | --- |
| **Figure A1:** Top row: boxplots of all strain types across all four intervertebral discs, including AL-bent discs (Disc 1 and Disc 2) and neutral discs (Disc 3 and Disc 4). Bottom row: strain distributions in the anterior-right (AL) and posterolateral left (PL) regions of the  AL-bent discs (Disc 1 and Disc 2). |  |
