# Supplementary material for: A Multiscale Ex Vivo Method to Investigate Intervertebral Disc Strain and Fiber Recruitment in Anterolateral Bending Using 9.4T MRI‐DVC and DIC Microscopy
Source: JOR Spine. 2025 Dec 25;8(4):e70154. doi: 10.1002/jsp2.70154 (PMC12740184; doi:10.1002/jsp2.70154)
Supplement: Supplementary file 2 — Appendix B: Representative images of each area of disc and corresponding crimp grade. [file JSP2-8-e70154-s002.docx]

Supplementary **Appendix B:** Representative images of each area of disc and corresponding crimp grade

|  |
| --- |
| **Figure B1:** Representative images from all four discs, each paired with its associated recruitment grade. Grades were defined as follows: 0 (uncrimped, fully recruited fibre); 1 (semi-recruited fibres, or if adjacent lamellae were fully crimped and uncrimped); 2 (fully crimped, not recruited). Each grade represents the mean grade from three images taken from the same region of the disc. |
